# Meningoencephalitis with *Streptococcus equi* Subspecies *equi* Leading to a Dural Arteriovenous Fistula

**DOI:** 10.1155/2021/9898364

**Published:** 2021-04-15

**Authors:** Jeroen Kerstens, Busra Durmus, Stijn Lambrecht, Ingrid Baar, Margareta M. Ieven, Thijs Van Der Zijden, Paul M. Parizel, Tomas Menovsky, Martin M. Y. Lammens, Philippe G. Jorens

**Affiliations:** ^1^Antwerp University Hospital, University of Antwerp, Department of Neurology, Edegem 2650, Belgium; ^2^AZ Middelheim, ZNA, Lindendreef 1, 2020, Antwerp, Belgium; ^3^Antwerp University Hospital, University of Antwerp, UZGent, UGent, Dept of Chemistry, 9000 Gent, Belgium; ^4^Antwerp University Hospital, University of Antwerp, Department of Critical Care Medicine, Edegem 2650, Belgium; ^5^Antwerp University Hospital, University of Antwerp, Department of Radiology, Edegem 2650, Belgium; ^6^Royal Perth Hospital, University of Western Australia, Medical School, Perth, WA, Australia; ^7^Antwerp University Hospital, University of Antwerp, Department of Neurosurgery, Edegem 2650, Belgium; ^8^Antwerp University Hospital, University of Antwerp, Department of Pathology, Edegem 2650, Belgium

## Abstract

Invasive infection with Lancefield group C streptococci in humans is extremely rare, with the vast majority of clinical isolates belonging to *Streptococcus dysgalactiae* subsp. *equisimilis*. We report a case of meningoencephalitis in a 69-year-old man caused by *Streptococcus equi* subsp. *equi,* a microbe that causes strangles in *Equus caballus* (i.e., the horse). This is only the fourth infection with this subtype of the central nervous system (CNS) reported in humans. The invasiveness of these bacteria, known to be capable of releasing strongly immunogenic exotoxins, is illustrated by white matter lesions that are present in the acute phase. This patient initially recovered well after treatment with antibiotics and glucocorticoids. However, the patient was readmitted 5 months later with multiple intraparenchymatous cerebral haemorrhages. Cerebral angiography confirmed the presence of a suspected superficial dural arteriovenous fistula (DAVF), which is seldom reported after CNS infection. The invasiveness of these bacteria was illustrated by white matter lesions present in the acute phase and the occurrence of a de novo dural arteriovenous fistula in the follow-up period.

## 1. Introduction

Lancefield group C streptococci (GCS) are a serological subgroup of beta-hemolytic streptococci [1]. Two of the 5 subspecies belong to the normal commensal flora of the human upper airway and are frequently asymptomatic colonizers of the skin, gastrointestinal tract, and female genital tract. The latter three (*S. equi* subsp. *zooepidemicus*, *S. equi* subsp. *ruminatorum*, and *S. equi* subsp. *equi)* are part of the commensal flora of the upper respiratory tract of horses, where they also cause various respiratory tract infections, and, in the case of *S. equi* subsp. *equi*, a highly contagious suppurative lymphadenitis (strangles) occurs [2]. Invasive GCS infection in humans is rare. Sporadically implicated in soft tissue infections and pharyngitis, the vast majority of clinical isolates belong to *S. dysgalactiae* subsp. *equisimilis* [3].

We report a unique case of human meningoencephalitis by *S. equi* subsp. *equi,* which was further complicated by occurrence of a de novo dural arteriovenous fistula (DAVF).

## 2. Case Presentation

A 69-year-old Caucasian male was admitted to the emergency department of our hospital after returning from a one-month vacation to Myanmar that included horseback riding. The patient had not taken malaria prophylaxis and did not receive any vaccinations prior to his departure. The patient's medical history was remarkable for gout, ciguatera, and neurosurgical reconstruction of the skull after a basilar skull fracture. The latter occurred 5 years before this event (after a fall from a horse), and it was complicated by cerebrospinal fluid (CSF) leakage and right-sided glossopharyngeal and vagal nerve damage, which resulted in right-sided vocal cord paralysis with hoarseness and sporadic aspiration. The patient did not take any medications and did not smoke, but he drank wine regularly.

For the last few days, the patient had been increasingly tired and somnolent, with complaints of headache, left-sided ear pain, and night sweats. One day prior to admission, the patient had become increasingly confused. On the day of his admission, he was found by his wife lying on the floor of his home, confused, agitated, shivering, and incontinent of urine. At the emergency department, the patient's temperature was 39.2 °C, and his heart rate was 111 beats per minute, with a respiratory rate of 20 breaths per minute and a blood pressure of 161/88 mmHg. The patient's peripheral oxygen saturation was 87%, and his Glasgow Coma Scale was scored as 9 (E2 M5 V2). Clinical examination revealed fasciculations of the face and lower legs and crepitation over the right lower lung on pulmonary auscultation. There was no purpura or nuchal rigidity.

At that time, blood results revealed a leukocyte count of 34 900 × 10^9^/l (normal: 4 300–10 000) (>95% being neutrophils) and a C-reactive protein (CRP) level of 204 mg/l (normal value <3). A chest X-ray revealed no abnormalities. A CT scan of the brain showed only nonspecific white matter lesions but no haemorrhage or oedema. Lumbar puncture examination revealed a leukocyte count of 1160/mm^3^ (almost all neutrophils), a normal glucose level, and a total protein level of 128 mg/dl (normal value 15–40) accompanied by an elevated lactate level of 6.1 meq/l. Gram stain showed irregularly shaped cocci. Bacterial meningitis was suspected, and after obtaining two sets of blood cultures, empirical therapy with 2 g of intravenous ceftriaxone every 12 h, 2 g of ampicillin every 4 h and 10 mg/kg of acyclovir every 8 h was initiated. Shortly after starting the antibiotic therapy, the patient became more somnolent and developed a left-sided hemiplegia, left-sided central facial palsy, and right-sided head and eye deviation, and the patient was admitted to the intensive care unit (ICU). Because of increasingly obstructive respirations and an increasing oxygen need by the next day, the patient was intubated and ventilated.

**Figure 1:**
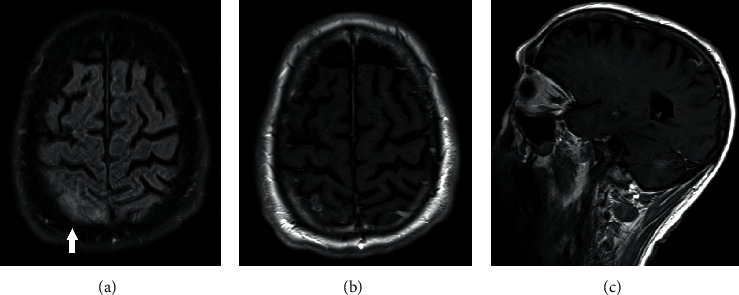
Fluid-attenuated inversion recovery images (FLAIR) showed a “dirty CSF” appearance in the right parietal region ((a), white arrow). Axial (b) and sagittal (c) T1-weighted images after the intravenous administration of gadolinium-chelate revealed a focal area of superficial contrast enhancement overlying the cerebral cortex, presumably representing a thickened pia mater.

Cerebrospinal fluid (CSF) cultures showed *β*-hemolytic colonies; blood and CSF cultures were positive for *Streptococcus equi*. Intravenous treatment with 2 g of ceftriaxone every 12 h was continued for 21 days, while ampicillin and acyclovir were discontinued. In vitro testing confirmed a sensitivity to ceftriaxone. Fluid-attenuated inversion recovery images (FLAIR) showed a “dirty CSF” appearance in the right parietal region (Figure 1(a), white arrow). Axial (Figure 1(b)) and sagittal (Figure 1(c)) T1-weighted images after the intravenous administration of gadolinium-chelate revealed a focal area of superficial contrast enhancement overlying the cerebral cortex, presumably representing a thickened pia mater and opacification of the left mastoid. In view of the MRI lesions and the left hemiplegia, one gram of intravenous methylprednisolone once daily was added to the treatment for 3 days with gradual tapering afterwards because meningoencephalitis with accompanying cerebral vasculitis was suspected [4]. After 4 days, the patient was weaned from the ventilator and extubated. Initially, the patient remained very agitated and disoriented and was temporarily treated with 25 mg of twice daily quetiapine and 100 mg of daily trazodone. Nine days after admission to the ICU, the patient was transferred to the neurology department. Later, this *Streptococcus equi* was identified as *S. equi* subspecies *equi* (at the National Reference Center for Invasive Non-Group B Beta-Hemolytic Streptococci, Belgium).

The patient initially recovered completely and had no complaints apart from mild gait disturbances and light-headedness at a follow-up appointment 3 months after discharge. Because of the patient's prior medical history of basilar skull fracture and opacification of the left mastoid on brain MRI, despite a lack of clinical evidence of acute otitis or mastoiditis, an additional CT scan of the base of the skull was performed to rule out possible portals of entry for central nervous system (CNS) infection. The CT scan revealed only an asymmetrical widening of the left squamosal suture; therefore, we opted for a conservative approach.

**Figure 2:**
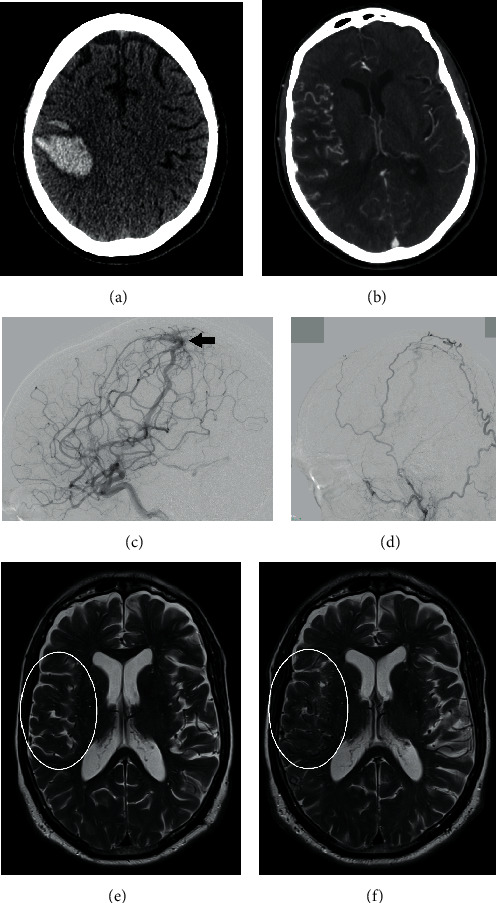
During readmission five months after the initial referral, consecutive CT scans showed multiple right-sided cerebral haemorrhages, the largest being located precentrally in the frontal lobe ((a), para-axial nonenhanced). CT angiography examination ((b), para-axial MPR) showed multiple tortuous, engorged pial veins of the right hemisphere. Cerebral angiography confirmed the presence of a suspected superficial dural arteriovenous fistula ((c), lateral projection of right internal carotid artery injection, large black arrow) in the right parietal region. The arterial feeders were mainly meningeal branches originating from the right ophthalmic artery and transosseous feeders from the right superficial temporal and occipital arteries ((d), lateral projection right external carotid artery injection); in addition, there was limited arterial supply to the fistula from the left middle meningeal artery, across the midline (not shown). Venous drainage was through a large, right temporal draining vein (large white arrow), with reflux into multiple tortuous, superficial frontotemporal veins, as shown in Figure 2(b), indicating a fistula with a high risk of haemorrhage. Comparison of axial T2-weighted images of MRI examinations at initial admittance (e) and at the second admission (f) showed the new finding of dilated pial veins (encircled), demonstrating the de novo development of the dural AV fistula.

One month later (5 months after the initial referral), the patient was readmitted with intracranial bleeding, and consecutive CT scans showed multiple right-sided cerebral haemorrhages, with the largest located precentrally in the frontal lobe with small perilesional parietal and temporal haemorrhages accompanied by a left hemiparesis and aphasia (Figure 2(a)). CT angiography examination (Figure 2(b), para-axial MPR) showed multiple tortuous, engorged pial veins of the right hemisphere. Cerebral angiography confirmed the presence of a suspected superficial dural arteriovenous fistula (Figure 2(c), lateral projection of right internal carotid artery injection, large black arrow) in the right parietal region. The arterial feeders were mainly meningeal branches originating from the right ophthalmic artery and transosseous feeders from the right superficial temporal and occipital arteries (Figure 2(d), lateral projection right external carotid artery injection); in addition, there was limited arterial supply to the fistula from the left middle meningeal artery across the midline (not shown). Venous drainage was through a large, right temporal draining vein (large white arrow) with reflux into multiple tortuous, superficial frontotemporal veins, as shown in Figure 2(b), indicating a fistula with high risk of haemorrhage.

Comparison of the axial T2-weighted images of MRI examinations at initial admittance (Figure 2(e)) and at the second admission (Figure 2(f)) showed the new finding of dilated pial veins (encircled), demonstrating de novo development of the dural AV fistula.

**Figure 3:**
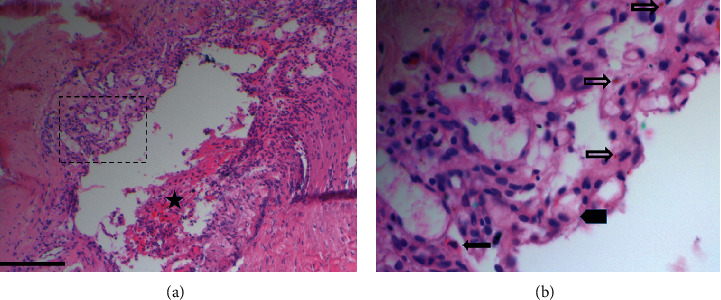
Histopathology of the excised dura with lymphocytic inflammatory remnants around a damaged venous sinus (a). In the upper part of the vessel wall (see also insert (b)) there is granulation tissue with many newly formed capillaries, lymphocytes, macrophages (thick arrow), a few eosinophils (thin black arrow) and some iron debris (thin open arrow). (Haematoxylin-Eosin Staining, bar = 1 mm; asterisk = peripheral blood).

The dural AV fistula was excised by the neurosurgeon. Microscopy of the resected dura showed a previously damaged vein with inflammatory remnants and granulation tissue in its wall (Figures 3(a) and 3(b)). The patient was discharged to home with a hemiparesis that was still present. Seven months later, after this second event, the patient's GCS was 15/15, and the hemiparesis had almost completely disappeared.

## 3. Discussion

*S. equi* subsp. *equi* infects the horse in which it causes a contagious disease called strangles [5]. Transmission occurs from horse to horse and indirectly by contact with water troughs, feed buckets, stalls, trailers, and grooming equipment. Flies can also act as vectors [5].

This microbe produces immunogenic capsular proteins, i.e., the SeM protein, which inhibits the ability of neutrophils to phagocytose bacteria [6]. This protein indeed binds fibrinogen and immunoglobulin G to inhibit the deposition of C3b, which further causes phagocytes to be destroyed [7]. *S. equi* is also able to release streptolysin S and streptokinase, further damaging cell membranes [8]. Several important genes involved with *S. equi* pathogenesis are also found in *S. pyogenes*, known to cause severe central nervous destruction by releasing exotoxins [9]. These immunogenic factors certainly contributed to the “cerebritis” as seen in this patient.

Occlusive arterial vasculitis or septic cortical thrombophlebitis may accompany bacterial meningitis and meningoencephalitis [10], with focal neurological signs because of necrosis of the cerebral tissue. Inflammation and thrombosis of both arteries and small cortical veins may be caused either by the accumulation of inflammatory cells between the endothelium and the internal elastic lamina of small arteries (to the point of obliteration of the lumen) or by the direct invasion of blood vessel walls by organisms. Autopsy data obtained from patients with pneumococcal meningoencephalitis indicate that perivascular or nodular inflammatory muffs appear in the white matter, accompanied by obliterating vasculitis, glial satellitosis, neuronophagy, and foci of demyelination of varied and irregular sizes [11]. We postulated that the intraparenchymal lesion documented on the diffusion-weighted MRI of our patient reflected ischaemia with cytotoxic oedema secondary to necrotising vasculitis [12, 13]. Although some of the improvement may have been caused by the evolution under antibiotic treatment, the administration of glucocorticoids, as proposed in isolated cases of cerebral parenchymal injury, such as cerebral vasculitis meningoencephalitis, certainly contributed to the recovery [4]. Remarkably, 5 months after the initial infection, a de novo dural arteriovenous fistula (DAVF) was observed at the site of the initial lesions and presented as a parenchymal haemorrhage. Dural arteriovenous fistulas (DAVFs) are a rare type of acquired intracranial vascular malformations consisting of the creation of a pathological shunt but are most commonly found in the region of the transverse, sigmoid, and cavernous sinuses, as was the case in our patient [14, 15]. DAVFs are typically supplied by meningeal arteries and exhibit venous drainage either directly into the dural venous sinuses or via cortical and meningeal veins. The aetiology of these lesions remains controversial. Although the causal role of venous thrombosis is supported by the association of DAVFs with hypercoagulable states and hormonal causes, a history of intracranial surgery, radiation exposure, pregnancy, trauma, or infection have all been proposed as sources of DAVFs [14, 15]. DAVFs have rarely been reported months after prior viral meningitis and bacterial meningitis [16, 17]. In our case, we assumed that destruction by these aggressive exotoxins and local thrombosis caused a profound inflammatory response in the brain that further evolved into symptomatic DAVF. Remnants of inflammation and reparation signs were still present in a dural vein when excision was performed.

A human CNS infection caused by GCS is very rare, with fewer than 50 cases reported in the literature [1,18–26]. Almost all cases of bacterial meningitis are caused by subspecies of *zooepidermicus*; complications have occurred in these cases, which are most commonly distant infection foci, such as endophthalmitis and endocarditis. Considering the *S. equi* subspecies *equi*, only a few cases were reported as an invasive infection in humans, and only three (apart from ours) involved the CNS [19, 23, 26]. In these cases, direct contact with horses could be pinpointed. To the best of our knowledge, our patient presented here is only the fourth reported case of human CNS infection with *S. equi* subsp. *equi*, and this patient is only the second case in an adult patient and the first reported case of meningoencephalitis with a DAVF.

The first case was published in 2003 by Elsayed et al. [19]. They reported a 13-year-old Canadian boy who lived on a horse farm with a medical history of resection of a petrous intracranial inflammatory mass at age 9; he presented with a 2-day history of fever, increasing neck stiffness, headache, anorexia, nausea, vomiting, lethargy, photophobia, bilateral deafness, and ataxia. The patient was diagnosed with bacterial meningitis that was empirically treated with cefotaxime and vancomycin, and the patient was switched to penicillin G after CSF cultures were positive for a multisensitive *S. equi* subsp. *equi*. The patient developed noncommunicating hydrocephalus that required ventriculoperitoneal shunting but recovered with only a mild residual ataxia and severe bilateral sensorineural hearing loss for which he received a cochlear implant. Popescu et al. [23] later reported a second case of a 75-year-old female admitted with fever and stupor who was successfully treated with 10 days of intravenous ceftriaxone and dexamethasone. She later stated that her neighbours were horse owners and she had visited their farm within two weeks prior to her admission. Very recently, a 13-year-old-boy with systemic lupus erythematosus who presented with sepsis and meningitis after contact with a sick pony has been described [26]. Although he recovered fully following eight weeks of intravenous ceftriaxone and oral rifampicin, the clinical course was complicated by a subdural empyema requiring neurosurgical evacuation [26].

In our patient, recent contact with infected horses in Myanmar was presumably the cause of the disease, and prior surgery was a possible route of entry.

In summary, we report a rare case of *S. equi* subsp. *equi* meningoencephalitis that initially recovered well after treatment with antibiotics and glucocorticoids. The cerebral lesions seen on MRI not only caused meningitis but also meningoencephalitis, and the postinfectious dural AV fistula accompanied by intraparenchymal bleeding illustrated the aggressive nature of the toxins released by this bacterium with a further inflammatory reaction.

## Data Availability

The data used to support the findings of this study are included within the article.

## Abbreviations

CT: Computed tomography
CSF: Cerebrospinal fluid
CNS: Central nervous system
DAVF: Dural arteriovenous fistula
GCS: Group C Streptococci.

## Supplementary Materials

Supplementary Materials Figure S1: timeline of the case.
