# Supplementary material for: Meningoencephalitis with Streptococcus equi Subspecies equi Leading to a Dural Arteriovenous Fistula
Source: Case Rep Neurol Med. 2021 Apr 15;2021:9898364. doi: 10.1155/2021/9898364 (PMC8272663; doi:10.1155/2021/9898364)
Supplement: Supplementary Materials — Figure S1: timeline of the case. [file 9898364.f1.ppt]

## Slide 1
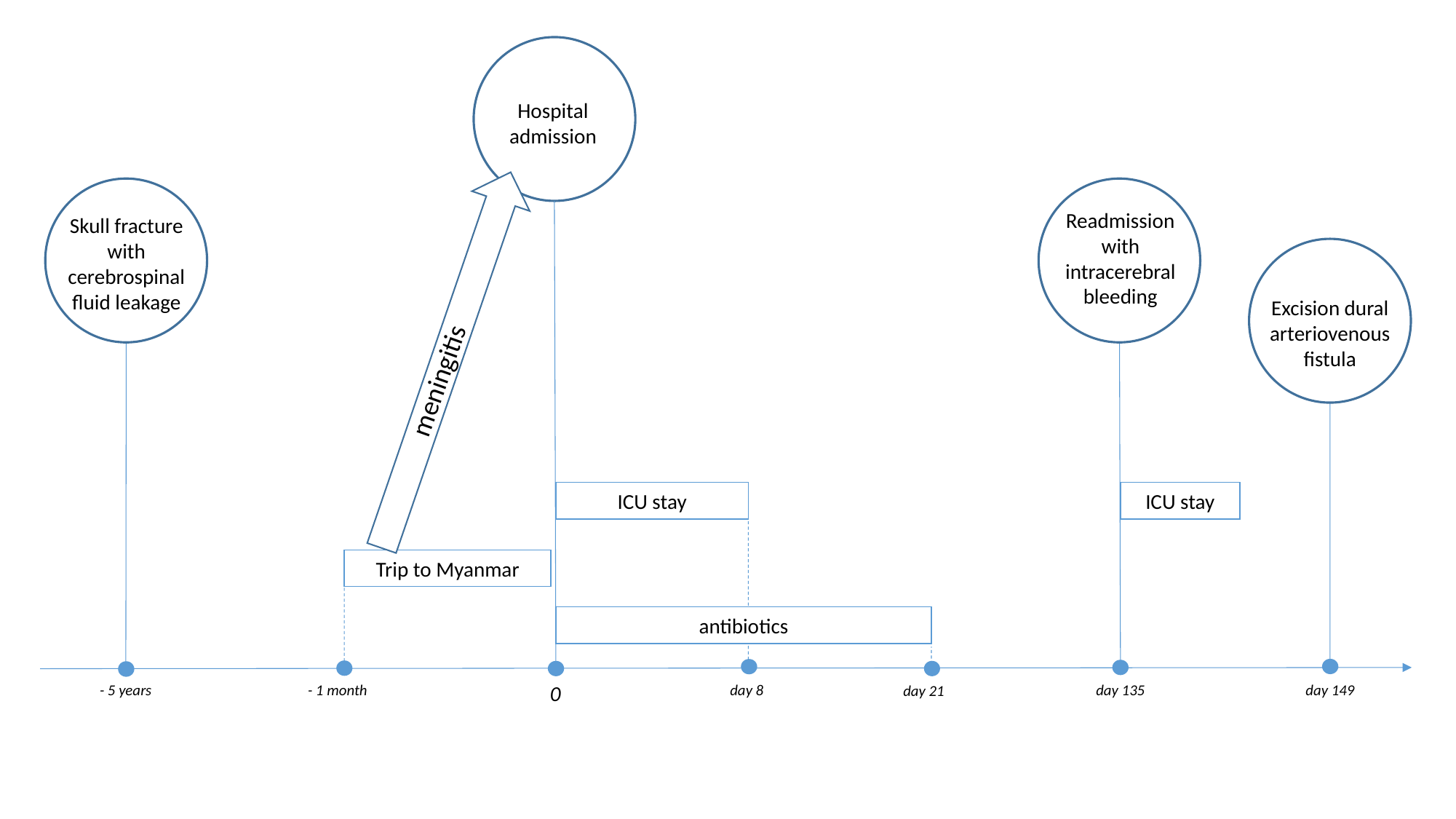

Hospital admission
Readmission with intracerebral bleeding
Skull fracture with cerebrospinal fluid leakage
Excision dural arteriovenous fistula
meningitis
ICU stay
ICU stay
Trip to Myanmar
antibiotics
- 5 years
- 1 month
0
day 8
day 135
day 149
day 21
